# Supplementary material for: Modeling of injury severity of distracted driving accident using statistical and machine learning models
Source: PLoS One. 2025 Jun 16;20(6):e0326113. doi: 10.1371/journal.pone.0326113 (PMC12169579; doi:10.1371/journal.pone.0326113)
Supplement: Attachment — Submitted filename: Response to Reviewers.docx [file pone.0326113.s002.docx]

***Reply to Academic Editor and Reviewer’s Comments***

Reply to the reviewer’s comments on the manuscript entitled ***‘*Modeling of Injury Severity of Distracted Driving Accident Using Statistical and Machine Learning Models*"***, by Neero Gumsar Sorum and Martina Gumsar Sorum. **Thanks to the Academic Editor, “PLOS ONE)” for allowing us to improve the current version of the manuscript and resubmission after revision. The authors would also like to sincerely thank all the anonymous reviewers for their encouraging and constructive comments to improve the quality of the manuscript.**

**Comments from the Academic Editor and Reviewers:**

Academic Editors Comments to Authors

1) Please ensure that your manuscript meets PLOS ONE's style requirements, including those for file naming.

**Reply:**

**The revised manuscript is prepared as per the PLOS ONE's style requirements, including those for file naming.**

2) a. For studies reporting research involving human participants, PLOS ONE requires authors to confirm that this specific study was reviewed and approved by an institutional review board (ethics committee) before the study began. Please provide the specific name of the ethics committee/IRB that approved your study, or explain why you did not seek approval in this case.

Once you have amended this/these statement(s) in the Methods section of the manuscript, please add the same text to the “Ethics Statement” field of the submission form (via “Edit Submission”).

For additional information about PLOS ONE ethical requirements for human subjects research, please refer to <http://journals.plos.org/plosone/s/submission-guidelines#loc-human-subjects-research>.

b. Please provide additional details regarding participant consent. In the ethics statement in the Methods and online submission information, please ensure that you have specified (1) whether consent was informed and (2) what type you obtained (for instance, written or verbal, and if verbal, how it was documented and witnessed). If your study included minors, state whether you obtained consent from parents or guardians. If the need for consent was waived by the ethics committee, please include this information.

If you are reporting a retrospective study of medical records or archived samples, please ensure that you have discussed whether all data were fully anonymized before you accessed them and/or whether the IRB or ethics committee waived the requirement for informed consent. If patients provided informed written consent to have data from their medical records used in research, please include this information.

**Reply:** **Not Applicable.**

3) We note that you have indicated that there are restrictions to data sharing for this study. PLOS only allows data to be available upon request if there are legal or ethical restrictions on sharing data publicly. For more information on unacceptable data access restrictions, please see <http://journals.plos.org/plosone/s/data-availability#loc-unacceptable-data-access-restrictions>.

Before we proceed with your manuscript, please address the following prompts:

a) If there are ethical or legal restrictions on sharing a de-identified data set, please explain them in detail (e.g., data contain potentially identifying or sensitive patient information, data are owned by a third-party organization, etc.) and who has imposed them (e.g., a Research Ethics Committee or Institutional Review Board, etc.). Please also provide contact information for a data access committee, ethics committee, or other institutional body to which data requests may be sent.

b) If there are no restrictions, please upload the minimal anonymized data set necessary to replicate your study findings to a stable, public repository and provide us with the relevant URLs, DOIs, or accession numbers. For a list of recommended repositories, please see

<https://journals.plos.org/plosone/s/recommended-repositories>. You also have the option of uploading the data as Supporting Information files, but we would recommend depositing data directly to a data repository if possible.

We will update your Data Availability statement on your behalf to reflect the information you provide.

**Reply:**

**Actually, we couldn't understand this statement properly at the time of article submission. Now understood, and the dataset used for the present study is uploaded as a supporting information file.**

Reviewers Comment to Authors:

Reviewer 1

\## This paper examines the individual and interacted effects of the influential factors on the injury severity of the DDAs using the Binary Logistic Regression (BLR) method, and to select the best machine learning (ML) model in predicting the injury severity of the DDA. The study is based on the 10-year accident data from India. Overall, the paper is well-organized and easy to follow. However, I have the following concerns.

**Thanks for the encouraging comments. The concerns in the manuscript are incorporated point by point and given below.**

*Major*

1) The dataset consists of approximately 78.6% non-fatal accidents and 21.4% fatal accidents, suggesting that a classification model could easily achieve an accuracy of at least 0.78 by simply predicting non-fatal accidents. However, the model in the paper yields an accuracy ranging from 0.4 to 0.77. Could the authors provide a more detailed explanation of these results, particularly regarding why the model does not perform as expected given the huge class imbalance?

**Reply:**

**In the context of imbalanced datasets, accuracy is often not the best measure of model performance, as it can be misleading. A model might predict the majority class most of the time and still achieve a high accuracy while performing poorly on the minority class (fatal accidents). Therefore, F1-Score and AUC metrics were also used for the evaluation of model performance in the present study. These metrics provide a more detailed understanding of how well the model is distinguishing between fatal and non-fatal accidents, especially in the case of imbalanced data. The possible reason for the actual performance of the ML model (accuracy =0.4 to 0.77) in the present study might be due to overfitting, underfitting, or a lack of variables that effectively capture the key differences between the non-fatal and fatal classes.**

2). The age groups are categorized as follows: below 18, 18-24, 25-40, and above 40. The second threshold is set at 24 (years old), which seems somewhat arbitrary. Could the authors clarify why this specific threshold was chosen? Furthermore, the third age group (25-40) contains the largest number of samples, which may lead to the dominance of this group in the analysis. This could skew the results. Could the authors consider either adjusting the groupings or discussing this potential bias?

**Reply:**

**It was planned to categorize the age group variable into teen age group (below 18), young age group (18-30), middle-aged group (31-45), and old age group (above 45) for the study. however, after analyzing, it was difficult to make group of middle-aged group data because young age group and old age group had higher numbers in the original data received (the dataset used in the present study is only for distracted driving: one cause of accident). Therefore, the age grouping was re-arranged into below 18, 18-24, 25-40, and above 40 so that each group could make a more or less balanced representation.**

*Minor:*
1) The study concludes that the "gender variable was not statistically significant to the injury severity of the DDA," which contrasts with findings from other studies. Could the authors provide an explanation for this discrepancy? It would be helpful to understand why gender does not appear to be a significant factor in this case.

**Reply:**

**The results of the present study indicated gender variable was not statistically significant to the injury severity of the DDA (p-value > 0.05). The statistical insignificance indicates that gender does not have a measurable impact on the injury severity of DDA. The possible explanation may be that other variables (e.g., age, vehicle type, nature of accident, and time of accident) played a much larger role in injury severity, and their effects might overshadow the potential impact of gender. When multiple factors are accounted for in the model, gender may not show any significant relationship. Another possible reason might be due to the imbalanced dataset (e.g., predominantly one gender). It may lead to an inability to detect any significant differences.**

2) On Page 19, Line 525, the authors apply three different hyperparameter conditions: 10-fold cross-validation with training sets of 70%, 80%, and 90%. Could the authors clarify why 10-fold cross-validation was chosen here? Based on the earlier results, 15-fold cross-validation seemed to yield the highest accuracy.

**Reply:**

**The main objective of hyperparameter tuning (changing k-fold cross-validation and train ratio values) was to study the variation in model performance, accordingly, the best ML model will change. So, in the first set, the ML algorithms were implemented by changing k-fold cross-validation values (5-, 10-, and 15-FCV) at a train ratio value (TR = 0.7), and in the second set, the algorithms were trained at three different TR values (0.7, 0.8, and 0.9) at a 10-FCV. Further, the two sets used in the present study were completely independent. In other words, it means that the first set can be carried out after the second set (in that case, the first set will become the second set) and vice-versa.**

Reviewer 2

1) How did the author confirm that the accident cases used were caused by distracted driving? If this problem cannot be solved, the core of this research will be seriously challenged

**Reply:**

**According to the police-reported accident data (used in the present study), those accidents which occurred due to mobile use, talking with passengers, or looking away from the roadway, were commonly designated as distracted driving accidents (DDAs). According to the police officials, the officer in charge asked the passengers (if involved in the accident) or the drivers (who survived the accidents) about the cause of the accident (on the spot or during medical treatment). If it was due to mobile use while driving, chatting/talking, or looking away from the roadway, then that particular accident was designated as a distracted driving accident (DDA). For more clarity, the following sentences are incorporated in the revised manuscript and highlighted:**

“In the present study, the DDAs were defined as those accidents which occurred due to driver engagement in secondary tasks (mobile use, chatting and talking, or looking away from the roadway) other than driving.” **(line numbers: 303-305, page number: 14)**

“The dataset contained two DDA categories: Non-fatal (not resulting in death within one month of the accident) and fatal (resulting in death within one month of the accident).” **(line numbers: 308-310, page number: 14 )**

2) Although 20 categorical variables have been identified, I think there is no deep connection between the involved variables and distracted driving. Is it possible to add factors such as using a mobile phone, operating the car's display screen, feeling sleepy, chatting, etc.?

**Reply:**

**The categorical variables identified in the present study were not the causes of distracted driving but were the factors that might contribute to the injury severity of the DDAs. Therefore, the main aims of the present study were (i) to examine the individual and interacted effects of these identified factors on the injury severity of the DDA using a logistic regression method, and (ii) to select the best ML model in predicting the injury severity of the DDA among trained six ML models.**

3) The machine learning model only uses the default settings of the Dataiku platform and does not fully explore the optimal parameter combinations.

**Reply:**

**By “The default settings of the Dataiku platform” statement, the authors meant that the ML algorithms available in the Dataiku platform were used, and no new algorithms were developed by their own in the platform. Regarding optimal parameter combinations, two sets of hyperparameter tuning were employed in the present study (mentioned in line numbers:…. And page number: …….):**

***In Set 1*, all six ML algorithms were implemented using 5, 10, and 15-fold cross-validation in a Train Ratio value of 0.70.**

***In Set 2*, the ML algorithms were trained at Train Ratio values of 0.70, 0.80, and 0.90 with 10-fold cross-validation.**

4) The performance of the model in dealing with the imbalance between the number of fatal and non-fatal accidents has not been deeply explored.

**Reply:**

**To deal with the imbalance between the number of fatal and non-fatal accidents, the present study employed a stratified k-fold cross-validation technique (instead of under-sampling and oversampling techniques) because this technique addresses this issue by ensuring that each fold contains approximately the same proportion of each class as the entire dataset. This is particularly important in imbalanced datasets where one class (e.g., non-fatal accidents) may be significantly more prevalent than the other (e.g., fatal accidents). By maintaining the class distribution in each fold, the stratified k-fold helps the model learn better representations for both classes and avoids bias toward the majority class**.

**This is described in section 3.6 of the revised manuscript (page 17).**

5) The time trend change of DDA data during 2011- 2020 has not been analyzed. Time series analysis can be added to study the variation law of accident severity over time and the dynamic influence of influencing factors.

**Reply:**

**The study of temporal stability/instability of contributing factors to the injury severity of DDA was not included in the present study and the same will be taken up in future research by the authors.**

6) Can the influence of environmental factors be considered?

**Reply:**

**There was no information about environmental factors in the police-reported accident data used in the present study, otherwise, these factors would have been included.**
