# Supplementary material for: Modeling of injury severity of distracted driving accident using statistical and machine learning models
Source: PLoS One. 2025 Jun 16;20(6):e0326113. doi: 10.1371/journal.pone.0326113 (PMC12169579; doi:10.1371/journal.pone.0326113)
Supplement: Attachment — Submitted filename: Response_to_Reviewers_auresp_2.docx [file pone.0326113.s003.docx]

***Reply to Academic Editor and Reviewer’s Comments***

Reply to the reviewer’s comments on the manuscript entitled ***‘*Modeling of Injury Severity of Distracted Driving Accident Using Statistical and Machine Learning Models*"***, by Neero Gumsar Sorum and Martina Gumsar Sorum. **Thanks to the Academic Editor, “PLOS ONE)” for allowing us to improve the revised version of the manuscript and resubmission after second revision. The authors would also like to sincerely thank all the anonymous reviewers for their encouraging and constructive comments to improve the quality of the manuscript.**

**Comments from the Academic Editor and Reviewers:**

Academic Editors Comments to Authors

\## Dear Dr. Sorum,

Thank you for submitting your manuscript to PLOS ONE. After careful consideration, we feel that it has merit but does not fully meet PLOS ONE’s publication criteria as it currently stands. Therefore, we invite you to submit a revised version of the manuscript that addresses the points raised during the review process.

**Reply:**

**The latest revised manuscript has been prepared by incorporating the concerns raised by the reviewers.**

Reviewers Comment to Authors:

Reviewer 1

\##

While the authors claim to have handled the imbalance using a stratified k-fold cross-validation technique, this reflects a misunderstanding of the method. Stratified k-fold ensures proportional representation of classes in each fold but does not actively address imbalance during model training.

Additionally, the machine learning models exhibit poor performance, even when considering F1-score and AUC. The results suggest that the models are performing only slightly better than random, indicating that they fail to effectively distinguish between non-fatal and fatal cases. Addressing the class imbalance properly and improving model robustness are necessary to enhance predictive performance. Without these improvements, the value of the ML approach remains questionable

**Reply:**

**Thank you for the insightful feedback. While it is true that stratified k-fold cross-validation ensures proportional representation of classes in each fold, we agree that it does not address class imbalance during model training. *The authors' claim may reflect a misunderstanding of the method’s limitations in handling imbalance in terms of model learning* - To address this, we could consider employing techniques like oversampling, undersampling, or using class-weight adjustments during training, which can more directly mitigate the impact of imbalance on model performance. Since the issue of imbalance in the dataset was not handled in the present study, this limitation is mentioned/addressed in the limitation section of the latest revised manuscript (the same is highlighted and incorporated in section 5.3 of the latest revised manuscript).**

*Major*

Reviewer 2

1) The authors have addressed all the comments point-by-point in their revised manuscript. The revised article now provides a detailed explanation of the DDA (distracted driving accident) determination criteria and data sources, which enhances the credibility of the study. The methodology is rigorous, with reasonable solutions proposed for addressing class imbalance and hyperparameter tuning. The authors have also acknowledged the limitations of the data (e.g., the absence of environmental factors) and expressed their intention to conduct future research on temporal trends. Overall, I have no further suggestions. It is recommended that the revised manuscript be accepted.

**Reply:**

**Thank you for your encouraging comments.**

***Note:***

While revising your submission, please upload your figure files to the Preflight Analysis and Conversion Engine (PACE) digital diagnostic tool, [https://pacev2.apexcovantage.com/](https://track.editorialmanager.com/CL0/https:%2F%2Fpacev2.apexcovantage.com%2F/1/010f01960dec91ec-0d34497e-a053-43cc-ae67-1d63f76e51c3-000000/P-hyY9h1TW-aAcrOx2jFzpPqV0IJjQ51PXCrgnZV30s=205). PACE helps ensure that figures meet PLOS requirements. To use PACE, you must first register as a user. Registration is free. Then, login and navigate to the UPLOAD tab, where you will find detailed instructions on how to use the tool. If you encounter any issues or have any questions when using PACE, please email PLOS at [figures@plos.org](mailto:figures@plos.org). Please note that Supporting Information files do not need this step.

***Reply:***

**Figs 1 and 2 were uploaded to the Preflight Analysis and Conversion Engine (PACE) digital diagnostic tool and checked.**
